# MRI and MRA Features of a Saccular Mycotic Aneurysm of the Cavernous Carotid Artery Resulting from Invasive Aspergillus Sinusitis

**DOI:** 10.5334/jbr-btr.886

**Published:** 2015-12-30

**Authors:** Mathieu Deltomme, Marc Schroeven, T. Morel Lawson, Jean-François Poma, Thierry Boulanger, Laurens JL De Cocker

**Affiliations:** 1 Assistant radiology, Department of Radiology, University Hospitals Leuven, Herestraat 49, B-3000 Leuven, Belgium; 2 Department of Pulmonology, Kliniek Sint-Jan, Kruidtuinlaan 32, B-1000 Brussel, Belgium; 3 Department of Neurosurgery, Kliniek Sint-Jan, Kruidtuinlaan 32, B-1000 Brussel, Belgium; 4 Department of Neurology, Kliniek Sint-Jan, Kruidtuinlaan 32, B-1000 Brussel, Belgium; 5 Department of Radiology, Kliniek Sint-Jan, Kruidtuinlaan 32, B-1000 Brussel, Belgium

## Introduction

Opportunistic infections are on the rise because of an increasing number of patients with immunosuppression and their prolonged survival [1,2]. Although invasive aspergillus sinusitis leading to a mycotic aneurysm of the intracavernous carotid artery is increasingly being reported, the magnetic resonance (MR) features supporting their mycotic origin are poorly known. A previous neuroradiological case report already extensively described the magnetic resonance imaging (MRI) and magnetic resonance angiography (MRA) findings of a fusiform aneurysm [3]. We now report the case of a saccular mycotic aneurysm involving the cavernous portion of the carotid artery, and discuss the imaging features supporting its mycotic origin.

## Case Report

**Figure 1:**
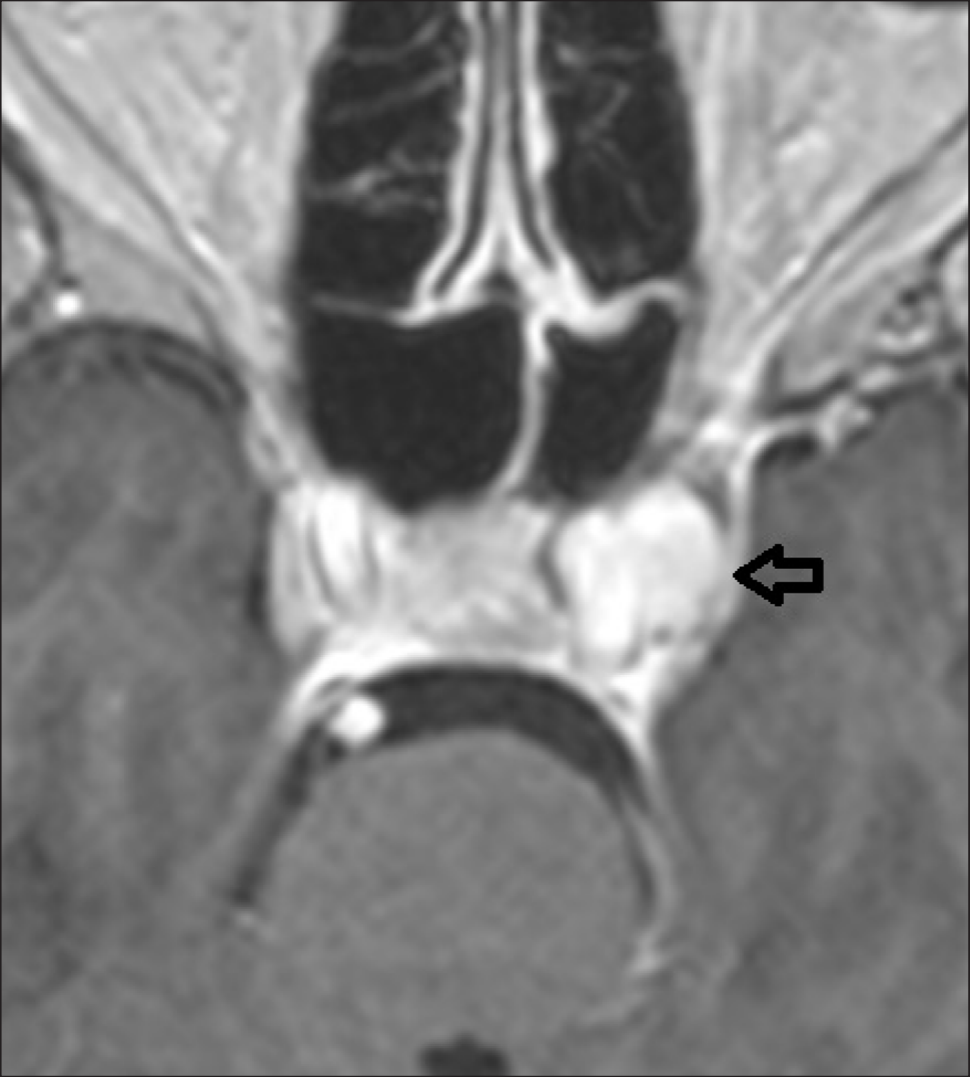
Contrast-enhanced T1WI: a contrast-enhanced and space-occupying structure in the left cavernous sinus (arrow), inseparable from the carotid artery.

**Figure 2:**
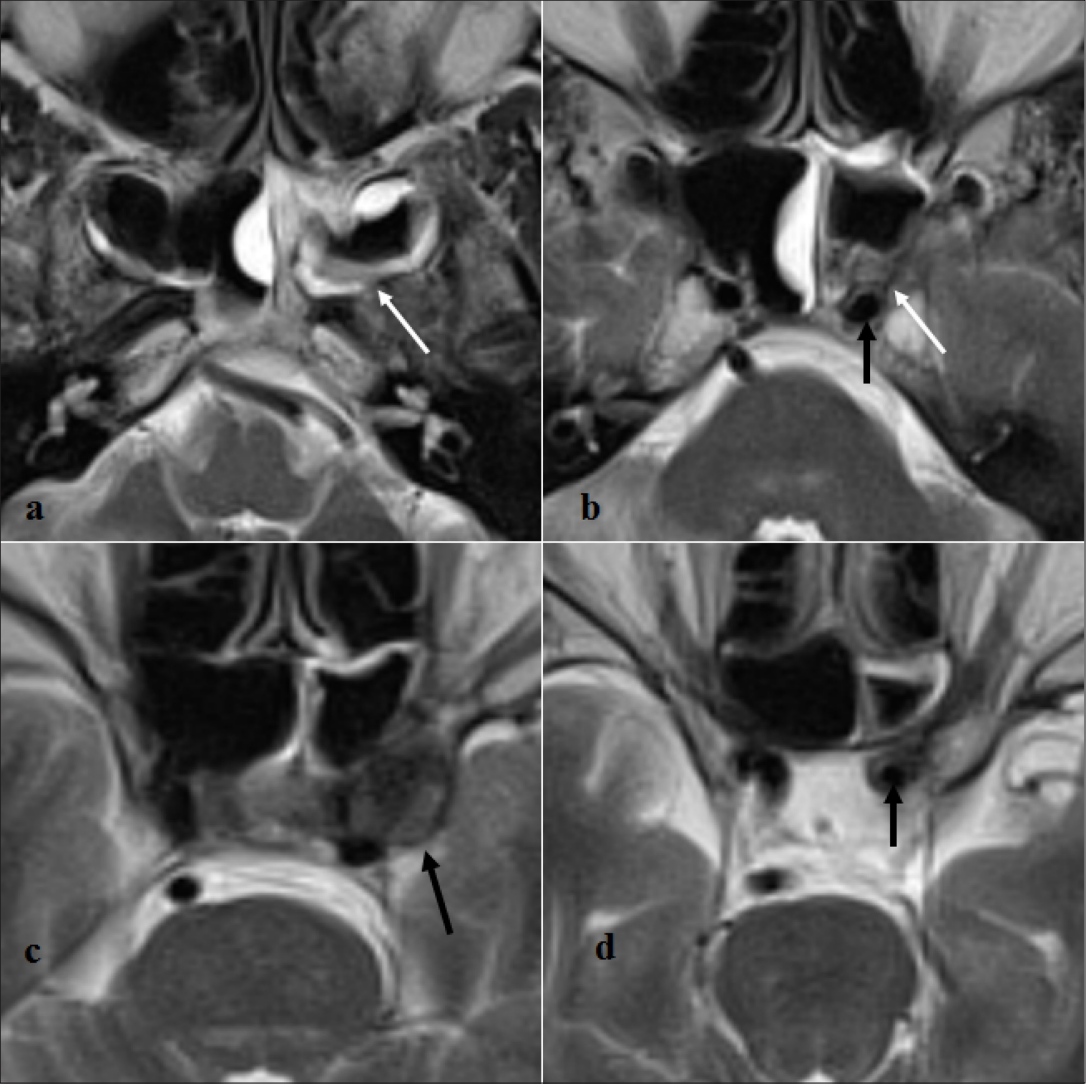
Axial T2WI at the level of the sphenoid sinuses, from caudal to cranial. **a,b)** wall thickening of both sphenoid sinuses, which appears T2-hypointense in the posterior aspect of the left sphenoid sinus only (white arrow). **b)** T2-hypointense tissue within the posterior aspect of the left sphenoid sinus (white arrow), inseparable from the left carotid artery, which shows discrete wall thickening immediately proximal to the aneurysm (black arrow). **c)** No flow voids are seen within the mycotic aneurysm of the left intracavernous carotid artery (black arrow). **d)** Concentric wall thickening of the left carotid artery is seen immediately distal to the aneurysm (black arrow).

**Figure 3:**
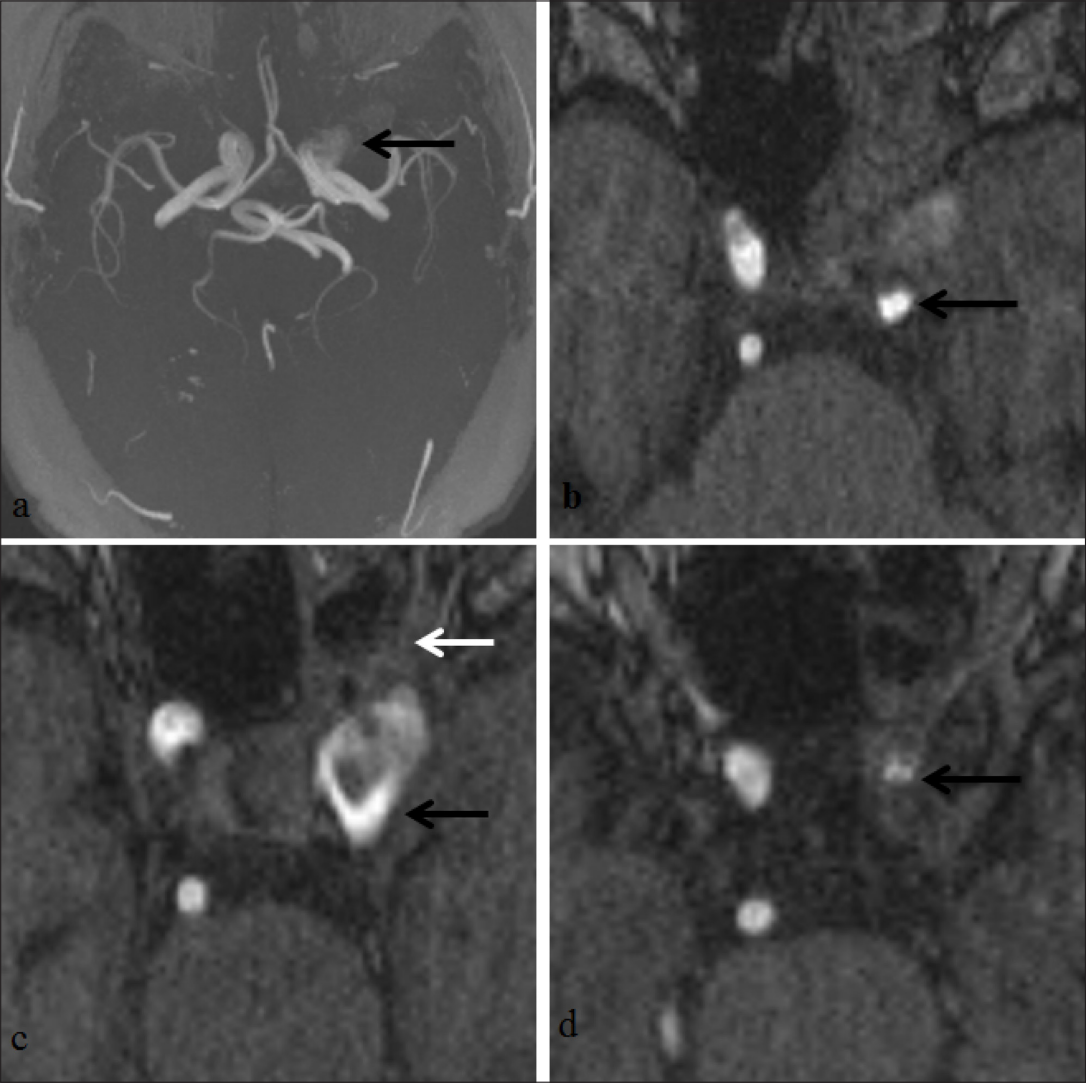
TOF-MRA, maximum intensity projection (MIP) (a) and axial source images from caudal to cranial. **a)** Intracavernous aneurysm (black arrow) with expansion toward the left. **b)** Stenosis of the internal carotid artery proximal to the aneurysm (black arrow) **c)** Intracavernous aneurysm of the left carotid artery (black arrow) with filling defects consistent with turbulent flow. The aneurysm is in continuity with inflammatory changes within the posterior aspect of the left sphenoid sinus (white arrow). **d)** Stenosis of the internal carotid artery distal to the aneurysm (black arrow).

A 67-year-old woman was admitted for retro-orbital and periorbital pain, increasing over 1 week and accompanied by progressive exophthalmia and palpebral ptosis on the left side. She was being treated for lung cancer metastasized to the liver and cerebellum. Physical examination revealed a complete ptosis of the left eyelid and complete absence of extraocular movements of the left eye, referable to the third, fourth and sixth cranial nerve. Head computed tomography (CT) showed mucous thickening of the left sphenoid and maxillary sinuses. Brain MRI revealed an expansive lesion involving the left cavernous sinus (Figure 1), which proved to be a new occurrence by comparison with a contrast-enhanced CT performed several weeks earlier. The lesion in the cavernous sinus appeared to be inseparable from T2-hypointense changes in the posterior part of the left sphenoid sinus (Figure 2a,b), whereas the remaining wall thickening of the ethmoidal, sphenoid and maxillary sinuses appeared to be smooth and T2-hyperintense (Figure 2d). The lesion in the cavernous sinus showed marked enhancement on contrast-enhanced T1WI and seemed to be inseparable from the carotid artery (Figure 1). Time-of-flight MRA (TOF-MRA) confirmed the presence of a saccular aneurysm of the cavernous portion of the left internal carotid artery, with evidence of turbulent flow within the aneurysm and with expansion of the aneurysm toward the left side (Figure 3a,c). In addition, circumferential wall thickening of the parent carotid artery was seen on T2WI immediately proximally (Figure 2b) and distally to the aneurysm (Figure 2d), with a corresponding concentric narrowing of its lumen visible on MRA. Endoscopic sinus surgery was performed with removal of mucus and fungal debris, which was confirmed as aspergilloma on pathologic examination. The diagnosis was made of a mycotic aneurysm of the intracavernous carotid artery resulting from local spread of invasive aspergillosis from the adjacent sphenoid sinus, secondary to an immunosuppressive status resulting from chemotherapy and longstanding systemic corticosteroid treatment. Because of the dismal prognosis of the malignancy and contraindication of antiaggregative drugs for reasons of a single hemorrhagic metastasis in the right cerebellar hemisphere, the multidisciplinary decision was made to refrain from endovascular treatment. The patient died a few weeks later.

## Discussion

The saccular (pseudo-)aneurysm described in this report did not appear to have any MRI or MRA features suggesting its mycotic origin, at least based on the configuration of the aneurysm itself. However, particular imaging features of the parent artery and surrounding structures in close vicinity to the aneurysm provided important clues toward its mycotic origin. First, the circumferential wall thickening restricted to the perianeurysmal parts of the carotid artery, not present earlier in time and in the absence of atherosclerosis elsewhere, could have suggested the presence of arteritis. Aspergillus arteritis may manifest with vessel wall thickening and stenosis or dilatation of the arterial lumen and with formation of pseudo-aneurysms in case of vessel perforation [4,5]. In addition, invasion of hyphae into the vessel lumen may cause in situ thrombosis, visible as absent flow void on conventional MR images, or filling defects on MR angiographic images (Figure 3) [4]. Although filling defects were indeed observed within the aneurysm on TOF-MRA (Figure 3), these were attributable to turbulent flow, as witnessed by the homogeneous contrast enhancement of the aneurysmal lumen (Figure 1). Second, the lesion in the cavernous sinus was seen in continuity with T2-hypointense inflammatory changes in the posterior recess of the sphenoid sinus (Figure 2a). The central location of the sphenoid sinus in the skull base is known to make the intracavernous carotid artery a predilection site for mycotic aneurysms secondary to invasive sinusitis [4]. In addition, our case nicely illustrates that the posterior part of the sphenoid sinus seems to be especially responsible for the intracavernous spread of aspergillus, which may be easily explained by its location immediately adjacent to the intracavernous carotid artery. Indeed, the inflammatory changes in the posterior sphenoid sinus were T2-hypointense and inseparable from the mycotic aneurysm, whereas the remainder of the inflammatory changes in the paranasal sinuses simply showed a smooth wall thickening, which was T2-hyperintense.

## Conclusion

The current case indicates how the mycotic origin of intracavernous carotid aneurysms may be deduced from perianeurysmal inflammatory changes rather than from the configuration of the aneurysm itself. In the studied case, circumferential vessel wall thickening of the carotid artery near the aneurysm, as well as its continuity with T2-hypointense inflammatory changes in the sphenoid sinus, was supportive of its mycotic origin. Further reports with detailed documentation of MR features are required to see if the latter is an invariable finding in additional cases.

## Competing Interests

The authors declare that they have no competing interests.
